# “We have been working overnight without sleeping”: traditional birth attendants’ practices and perceptions of post-partum care services in rural Tanzania

**DOI:** 10.1186/s12884-015-0445-z

**Published:** 2015-02-03

**Authors:** Gladys R Mahiti, Angwara D Kiwara, Columba K Mbekenga, Anna-Karin Hurtig, Isabel Goicolea

**Affiliations:** School of Public Health and Social Sciences, Department of Development Studies, Muhimbili University of Health and Allied Sciences, P.O. Box 65454, Dar es Salaam, Tanzania; School of Nursing, Muhimbili University of Health and Allied Sciences, Dar es Salaam, Tanzania; Division of Epidemiology and Global Health, Faculty of Medicine, Umeå University, Umeå, Sweden

**Keywords:** Perceptions, Post-partum care services, Practices, Tanzania, Traditional birth attendants

## Abstract

**Background:**

In many low-income countries, formal post-partum care utilization is much lower than that of skilled delivery and antenatal care. While Traditional Birth Attendants (TBAs) might play a role in post-partum care, research exploring their attitudes and practices during this period is scarce. Therefore, the aim of this study was to explore TBAs’ practices and perceptions in post-partum care in rural Tanzania.

**Methods:**

Qualitative in-depth interview data were collected from eight untrained and three trained TBAs. Additionally, five multiparous women who were clients of untrained TBAs were also interviewed. Interviews were conducted in February 2013. Data were digitally recorded and transcribed verbatim. Qualitative content analysis was used to analyze data.

**Results:**

Our study found that TBAs take care of women during post-partum with rituals appreciated by women. They report lacking formal post-partum care training, which makes them ill-equipped to detect and handle post-partum complications. Despite their lack of preparation, they try to provide care for some post-partum complications which could put the health of the woman at risk. TBAs perceive that utilization of hospital-based post-partum services among women was only important for the baby and for managing complications which they cannot handle. They are poorly linked with the health system.

**Conclusions:**

This study found that the TBAs conducted close follow-ups and some of their practices were appreciated by women. However, the fact that they were trying to manage certain post-partum complications can put women at risk. These findings point out the need to enhance the communication between TBAs and the formal health system and to increase the quality of the TBA services, especially in terms of prompt referral, through provision of training, mentoring, monitoring and supervision of the TBA services.

## Background

The post-partum period – defined by the WHO as the 42 days after childbirth [1]– is an important part in the continuum of care for improving maternal and child health [2-4]. This period is not as closely monitored and complications to both newborn infant and the mother i.e. bleeding, infections or post-natal depression can present without being promptly addressed [1]. Therefore adequate and timely post-partum care can lead to detection and management of such complications [3]. Also, this post- partum period may be an opportunity to not only to prevent maternal death, but also to maintain and promote the health of a woman and her baby.

Research shows that, in many low-income countries, formal post-partum coverage is much lower than skilled delivery coverage and antenatal care coverage [5] even though the promotion of healthy behaviors and care of the mother and the baby at the community and family levels are also important in this period [1,3]. Both social and clinical health-care services can be provided in the post – partum period including: management of anemia, promotion of nutrition and the provision of vitamin A supplementation, complete tetanus toxoid immunization if required, [1,3], provision of counseling and family planning education, breastfeeding support and provision of insecticide - treated nets [1,3].

Post-partum care is not only provided by health-care workers in health care services; it is also provided by the family and the community members [6]. In many low-income settings, post-partum care is to a large extent provided by relatives and Traditional Birth Attendants (TBAs) [7]. According to the WHO, a TBA is a person who assists a mother during childbirth and who initially acquired her skills by delivering babies herself or by working with other TBAs [8]. They are highly respected, trusted by their clients and conduct their practices within community settings [9-12]. However, studies also show that interventions directed towards training and involving TBAs are not useful and might be harmful, since they might discourage mothers to deliver at health facilities. TBAs cannot be considered skilled birth attendants and the care they provide should not curtail child and maternal mortality and morbidity [13-18].

The approach of health systems to the role of TBAs in maternal health-care strategies has been affected by policy changes [14]: from support for TBA training and clean delivery, policies moved towards skilled delivery and emergency obstetric care and in the later, TBAs were completely left out of the maternal health care programmes [19-21]. More recently, the continuum of care approach (launched in 2005) validated the relevance of TBAs’ specific skills, and asked for a coordinated response that connected them with the formal health systems [5]. This approach acknowledges that TBAs have the potential to improve maternal and newborn health at the community level. It emphasized their roles in caring for pregnant women and conducting deliveries, but not in solving complications [22].

In support of this, existing studies have demonstrated that in low-income countries TBAs might play a more relevant role during the post-partum period than formal services do. For example, a study done by Titaley et al. [23] revealed that TBAs have an important role during the post-natal period and were very well utilized and trusted in some communities [18]. Another example is highlighted in a quantitative study conducted in South Africa, where 76% of TBAs reported making post-natal visits to their clients [23].

### Post-partum care in Tanzania

Despite the high rates of Antenatal Care (ANC) use and increased institutional delivery in Tanzania, the coverage of post-partum services remains low [3,4,24-26]. Around 96% of pregnant women receive at least one ANC check-up, but only 43% receive the recommended four check-ups. In addition, only 51% of deliveries are attended by skilled health professionals, and PNC coverage drops to 35% [27]. Poor post-partum utilization might be related to social and cultural factors, amongst them the use of TBAs at home after women are discharged from health-care facilities with their babies.

Tanzania has made strong efforts to improve maternal health, as is evidenced in the numerous policies and guidelines addressing this issue [4]. In all these policies the community and informal birth attendants such as TBAs are mentioned as key stakeholders in improving maternal health [4,27]. Furthermore, the national health policy stipulates the intention of integrating TBAs through developing and coordinating services offered by TBAs and other traditional medicine practitioners [27]. Those policies do not support TBAs attending deliveries, but encourage them in engaging in other important roles such as counseling, detection of danger signs and provision of adequate referrals to formal health-care services [4]. Regarding post-partum care, the presentation of Reproductive and Child Health (RCH) card 4 mentions the post-partum services being offered in the health-care facilities. In addition, post-partum guidelines have recently been published, however their practicality and the integration of TBAs in post-partum care are not clearly stipulated. There are a number of studies focusing on the role of TBAs in antenatal care and delivery in Tanzania [28-35]. Despite the existence of many studies exploring TBAs’ involvement during pregnancy and delivery, there is a scarcity of research that explores TBAs’ practices and their perceptions during the post-partum period [28,36]. Therefore, the aim of the study was to explore TBA practices and perceptions of post-partum care in rural Tanzania. The findings will help in informing policy makers and other stakeholders about introducing proper interventions such as policies and guidelines in improving maternal and neonatal health through utilization of post-partum care services.

## Methods

### Study area

The study was conducted in Kongwa,one of the five districts in the Dodoma region. This study is part of a larger ongoing health system research project in the Dodoma region aimed at strengthening health systems for accessibility, equity and poverty alleviation in rural areas. The project explores both the supply side of human resources, including governance of the health system, equipment and supplies and the demand side including users and the community perspective on access to services. This study contributes to generation of knowledge on the demand side, through exploring the perspectives of different actors, namely women, their partners and community based health agents such as TBAs. Kongwa was selected because of its rural characteristics, since the focus of the entire project was to explore health systems’ in a rural setting, far from the capital city.

Kongwa has a population of 248,656, with 90% living in rural areas. The area is agricultural and people mainly engage in cultivation and livestock keeping. Its residents are also engaged in other activities such as trade and mineral extractions. The area has a poor transport system with poor roads, causing difficulties for women from rural areas in seeking health services including referrals.

There are a total of 46 health facilities in the district consisting of one district hospital, four health centers and 41 dispensaries. In the Dodoma region, 97.8% of pregnant women get at least one antenatal check-up, well short of the recommended four visits beneficial to all women [27]. There is no disaggregated data available in this region regarding the number of antenatal care visits that women receive. The available data is on national level indicating the percentages of mainland women utilizing ANC services in the rural areas:3.8% of women receive one visit, while 54.5% receive 2 – 3 visits, and 39.5% receive 4 or more visits. Institutional delivery in the Dodoma region drops to 45.9%, and only 33.8% of mothers receive post-natal check-ups, defined as check-ups of the mother and the child [22]. Kongwa district has engaged in a number of interventions aimed at improving maternal health, such as: 1) training service providers in Emergency Obstetric Care (EmOC), 2) implementing a waiting maternity home (chigonela) at the district hospital, where women who live far away or have complicated pregnancies can stay until delivery, 3) receiving equipment for facilities with help from developing partners such as delivery beds, and other equipment for conducting delivery such as gallipot and kidney dishes,4) motivating TBAs by paying to bring mothers to give birth at the facilities –the latter is currently stopped due to unavailability of funds from the government and 5) implementing community sensitizations on institutional delivery. The district has increased the number of health facilities from 42 in 2011 to 46 currently. The referral system has improved through the provision of mobile phones to all health facilities and securing of five ambulances for all four health centres and the district hospital.

### Participants’ recruitment and data collection

For this study, we conducted individual interviews with three different groups of participants. The first group comprised what we labelled as “untrained TBAs”. “Untrained TBAs” were those who are not linked in any way to the health-care system. They have not received any sensitization on health issues and they have not registered with the formal health care system. Eight individual interviews with “untrained TBAs” were conducted; they were approached by the researcher through the community leaders. The second group is comprised of what we called “trained TBAs”. “Trained TBAs” are registered and well known within the health-care system. They have received sensitizations on health issues from the formal health-care sector, have been advised to avoid attending deliveries and, in case they are faced with a delivery that cannot be referred, they have received information on aseptic techniques in order to conduct a clean delivery. Three trained TBAs participated, who were approached through those in charge of the health care facility.

Trained TBAs, but not untrained TBAs, have been given incentives to bring mothers to deliver in health care facilities. However, such incentives had stopped due to shortage of funds when this study was conducted. Both the trained and untrained TBAs’ occupation was agriculture, and they were all local residents and of an old age. It was emphasized that the researcher was not representing the government or the Ministry of Health and the findings would not be used against TBAs in Kongwa.

The third group comprised five multiparous women who have been clients of untrained TBAs. They were contacted through the untrained TBAs. Ages ranged from 27 to 54 years – mean age 40. Their parity ranged from 2 to 7 children (mean parity 5) and their main occupation was agriculture with a basic education of standard seven.

The inclusion criteria for untrained TBAs were those who have been conducting deliveries and were referred by the village leaders. Untrained TBAs were located by village leaders because, since they are knowledgeable of the structure of the village, know the TBAs and they were also easy to be identified and approached by the researcher. For the trained TBAs, the inclusion criteria were those who have been conducting deliveries and were referred by the health-care providers. For untrained TBAs’ clients, the inclusion criteria were the clients who were referred by the interviewed TBAs.

Data collection took place in February 2013. Participants were approached by the researcher, the aims of the study were explained and they were asked for their permission to conduct the interview and record it. The location of interviews for untrained TBAs and their clients were at their individual premises and for trained TBAs it was at the village office. The house where village meetings are conducted may be far away from the health services. All participants chose the location comfortable to them. The researcher made sure that no one else apart from the intended participants was there during the interview.

Interviews continued until saturation was reached, meaning no new information relevant to answer our research question was emerging [37]. All individual interviews followed an open format, and several aspects were explored, such as practices, perceptions and post-partum care in general. Across the interviews, TBAs and women used different words to refer to the practices that they conduct during the post-partum period and their perceptions of formal post-partum care. Direct translations of these terms will be maintained in the quotations, but elsewhere we will use the term “post-partum care” when referring to a period after child birth of up to 42 days.

The interviews were conducted in Kiswahili, which was the medium of communication of the researcher and all of the respondents. Transcriptions in Kiswahili were translated into English and entered into Open Code 3.4 for managing the data and facilitating the analysis [38].

### Data analysis

The digitally recorded individual interviews were transcribed verbatim. Coding was conducted with the Kiswahili transcripts in order to be close to the text. Three of the authors are fluent in Swahili and were involved in this process. Codes were then translated into English and for developing categories and themes all authors were involved. Data were analyzed using qualitative content analysis, focusing on aspects related to postpartum care services [39]. After reading the interview transcriptions several times meaning units that referred to postpartum care were identified. From the meaning units – short summarized versions of the sentences – codes were developed. Codes were grouped together to build categories, which reflected the manifest content, i.e. what the interview transcripts conveyed regarding post-partum care (see the example of the coding process in Table 1). During the process of merging codes into categories, similarities and differences between the three different groups of participants were also highlighted. The field notes were used during analysis so as to capture the points that were taken during the interviews. Additionally, notes were used during the coding process to enrich and triangulate the information conveyed in the interviews.

**Table 1:** **Example of the process of analysis from meaning unit to category**

**Meaning unit**	**Condensed meaning unit**	**Codes**	Category*
They might call me at night and I help her (post-partum mother), I sleep there and observe her (post-partum mother) condition and how she is progressing	Called at night to help a mother and sleep there to monitor progress	TBAs responsive to women	Doing close follow-ups and their proximity to post-partum mothers matter
		TBAs monitor women closely	

*The category was later part of the theme “Caring rituals and being close to women”.

### Methodological considerations

In evaluating trustworthiness in qualitative research, criteria such as credibility, transferability, dependability and confirmability can be used [39,40].

In understanding the subjects’ reality and their complex situations we applied purposive sampling in our study. Purposive sampling was used, meaning that informants were selected due to their ability to contribute to answering our research questions [39,41]. They were selected because of their experiences of either being TBAs or being post-partum mothers and having received care from the TBAs.

Village leaders assisted in selection of the informants after the researcher’s explanation on the objective of the study and the expected source of information. During the initial interviewing process, the researcher screened the informants based on the criteria and objective of the study and expected informants. The interviewing process continued after the researcher assured herself that the respondent met the criteria. We followed an emergent design: after each of the interviews, aspects that emerged and that we thought were relevant were included in the following interviews. Following an emergent design enhances dependability [37].

In order to enhance trustworthiness of the study the research team consisted of people with variety of professional background, including experts in sexual and reproductive health issues, public health and health system research.

The detailed description of the study context, selection criteria, data collection and analysis process was complemented by quotes to allow readers to judge the transferability of the findings. Presentations of preliminary findings to the research team allowed critique from an outsiders’ perspective, especially alternative interpretations, which contributes to credibility.

Interview guides underwent translation from English to Kiswahili, as the respondents were more comfortable in Kiswahili than in English. The original transcripts were analyzed in the Kiswahili language, which increased the credibility and dependability of our findings. During the data collection process, the researcher kept an open mind and tried to put her pre-understandings into brackets in order to ensure confirmability.

### Ethical considerations

Ethical approval to conduct this study was granted by the Senate Research and Publication Committee of Muhimbili University of Health and Allied Sciences (Ref. No. MU/ RP/AEC/Vol.XII/). Furthermore, permission to conduct this study was obtained from the Kongwa district executive director’s office, and lastly from the Kongwa district Medical Officer’s office. Informed consent was sought from each potential participant and names were not written anywhere in order to ensure confidentiality. They were all assured that information given would be treated with strict confidentiality.

## Results

From the analysis four themes and a number of related categories emerged that cross – cut categories and reflected the latent content and these are presented in Table 2. Our study found that TBAs take care of women during post-partum with rituals appreciated by women. It also shows that even if TBAs are ill-equipped to detect and handle post-partum complications they try to provide care for them, which could put the health of the woman at risk.

**Table 2:** **Emerging themes and related categories**

**S/N**	**Themes**	**Categories**
1.	Caring rituals and being close to women.	• Making close follow-ups and their proximity to post-partum mothers matter
		• Preparation of special food for post-partum mothers
		• Sponging of post-partum mothers
		• Offering services for charity
2.	Daring to provide care complications	• Managing complications using bare hands
		• Applications of traditional medicines for treating complications
3.	Referring under uncertainty of services’ quality and only when complications emerge	• Deciding to take women to the health facility if fail to handle complications
		• Post-partum services are used for baby’s clinic
		• Formal post-partum care: valued but at the same time uncertain about its quality
4.	Weak connections between TBAs and formal health-care services	• TBAs compare their knowledge with that of formal health systems
		• Acquire knowledge through experience and apprenticeship
		• There is a poor link to the formalized health-care systems

### Caring rituals and being close to women

The theme “Caring rituals and being close to women” refers to TBAs’ practices of post-partum care. In this theme both trained and untrained TBAs reported to practice favorable practices when providing care. During the interviews TBAs were asked about their mode of working after assisting with delivery, and they reported on the follow-ups they make and being close to their clients through visiting them. Such visits could last from three days until as long as a month. The most commonly expressed activity was for the TBAs to make follow-ups to monitor progress of the health of the post-partum mother, i.e. asking about pain or food intake. TBAs’ close follow-ups over a long period of time varied depending on the location of clients, meaning that women were followed up closely when the TBAs lived quite close to post-partum women. In this study both untrained and trained TBAs reported sponging the abdomen of postpartum mothers (but not genitals). It was indicated that they use hot water for sponging mothers as a pain relief intervention. These findings are supported by the women’s responses that the TBAs sponge them with hot and sometimes warm water.

“We elders (TBAs), we have been working overnight without sleeping monitoring a woman who has delivered and her baby, looking after the baby’s umbilical cord to see if it is making good progress.” (Untrained TBA No. 5)

“When sponging the mother who has delivered, I sponge the woman with hot water to remove the dirtiness in the abdomen.” (Untrained TBA No. 4)

“They (TBAs) have been helping mothers by sponging with warm water when massaging them.” (Client of untrained TBA No. 3)

Both groups of TBAs expressed that they prepare special food such as soup made from chickens or goats, porridge and potatoes when the women deliver. These were also commented on by the women who said that during the post-partum period the TBAs prepare certain food to give the post-partum mothers energy.

“Yes, I give her tea, porridge, food such as potatoes mmh (nodding head). Soft food.” (Untrained TBA No.1)

“… After delivery, I go there for cooking food for the postpartum mother. That’s it.” (Trained TBA No. 2)

Both groups of TBAs expressed that since they live a short distance from the women they attend, it became easy to serve them for a long time compared to the difficulty in reaching health facilities due to poor infrastructure. Women also responded on the issue of distance, where they reported that it was easier for the TBA to make close follow-ups after they delivered.

Both groups of TBAs reported that they perform these caring rituals on a voluntary basis, without demanding money from the women for them to be paid. However, the interviews also show that a certain amount of money was provided to TBAs for their services with respect to appreciation for the service they have offered for them to buy some stuffs such as soaps.

“Money is for buying soap, how can I wash my hands? The mother has to offer the money. She has to offer about seven thousands shillings. Eeeh! It’s like a charity to her.” (Untrained TBA No. 2)

“To me as a TBA, I am just helping her. Eeeh! If she has to thank me they will think about that. I have been doing all this but I cannot demand her to offer anything. She has to think for that on her own time.” (Trained TBA No. 1)

“Yes, you have to pay her. Sincerely she does not demand for the payments from the mother. It’s the mother herself that can decide to pay her. But she cannot demand.” (Client of untrained TBA No. 2)

### Daring to provide care during complications

Besides the rituals described above, TBAs also pointed out that they detected and handled complications and health problems that might emerge during post-partum, such as backache, fever and retained placenta. Both untrained and trained TBAs reported removing retained placenta with their hands, the difference being that trained TBAs might use gloves, while untrained TBAs reported using their bare hands.

“It means when the mother delivered and found that she had problems of retained placenta I just did some activities (drawing the retained placenta) there, I put my hand there, I cleaned inside the womb.” (Untrained TBA No. 2)

TBAs also described how they used traditional medicine. Untrained TBAs also described that they use herbal medicine to treat women such as “mgongola”, which is used for treating pains. Traditional herbal remedies were also provided with the aim of removing retained placenta from the uterus.

“For a post-partum mother, when she has delivered and has a retained placenta, she will be given herbs and it comes out.” (Trained TBA No. 2)

### Referring under uncertainty of services’ quality and only when complications emerge

This theme refers to TBAs and women’s perceptions of referral practices and the quality and availability of post-partum care offered within formal health-care services. Regarding referral practices, untrained TBAs mainly refer women when physical complications arise that cannot be handled by them such as post-partum hemorrhage (PPH). This differed from the trained TBAs, who expressed that they always advise post-partum women seek care at health-care facilities, although they also reported previously that they also provide care when women face retained products in the womb.

“If she (post-partum mother) is bleeding too much (PPH) you can’t help her and it is much better to rush her to the health-care facility.” (Untrained TBA No. 5)

“My work is to take a mother to the health facility. Eeeeh! When they inform me I advise them to come to the health facility where there are professionals, eeh ale (meaning yes).” (Trained TBA No. 2)

“Eeeh!I called the TBA to check for my problem (PPH) after childbirth. If she failed, eeeh! She instructed us (the mother and family members) to go together with her (TBA) to the health facility. We went together.” (Untrained TBA’s client No. 3)

Regarding the importance and availability of formal post-partum services for women after delivery, differences existed between the three groups. Untrained TBAs reported perceiving that women’s use of formal post-partum care was mainly for the baby’s clinic and not for themselves. They indicated that women normally bring their children to get vaccinations and weight measurement. Their responses on this issue differed from the trained TBAs as they reported on the need for formal post-partum visits not only for the babies, but also for post-partum mothers. Women’s responses also showed poor awareness of the importance and availability of post-partum care for them after delivery.

“Women after childbirth do not go back to the health facility if they had a safe delivery, they just continue with porridge and bathing at home. They stay for some time at home and later on bring the baby to the health facility.” (Untrained TBA No. 8)

“They (post-partum mothers) come back as they have been instructed to come back after childbirth; they (health workers) are professionals for detailed investigations.” (Trained TBA No. 1)

“Aaa, for a mother after childbirth at home, it is not important to use health-care services. It is important to bring the baby only.” (Client of untrained TBA No. 4)

### Weak connections between TBAs and formal health-care services

This theme refers to the sources of TBA training and the relationship between formal health-care services and TBAs. Both groups of TBAs had mixed responses about their ability to handle the women who have given birth. Untrained TBAs equated their knowledge with that of the health workers. They mentioned that they refer their clients to their colleagues (health facilities) where they can get further treatment. However, they considered that health facilities’ abilities to diagnose and handle the problems were superior to those of the TBAs. They also mentioned that their education was poor compared to that of the health workers. However, the trained TBAs perceived the connection to be good as they were invited to the health facilities to assist women during deliveries. Women’s expressions also complement those of trained TBAs; they also had less confidence in TBAs, especially when complications arise that the TBAs cannot handle.

“Aaah! There is no difference if she delivers safely at home. We have assisted most of them.” (Untrained TBA No. 1)

“After childbirth my work is to look after the baby and make sure the umbilical cord of the baby doesn’t bleed too much, eeeh! Then looking for mother’s health with my colleagues (health workers). They check (investigate) her health to see if it is progressing well.” (Untrained TBA No. 1)

“When a post-partum mother has a problem of too much bleeding in the body, they call a TBA and she will instruct that the post-partum mother has to go to the health facility and she will escort the mother. Eeeh!” (Client of untrained TBA No. 1)

Both trained and untrained TBAs reported on the lack of formal training in a formalized and organized way on post-partum care services. They said that they used to share the knowledge among themselves regarding delivery practices and caring of a mother during the post-partum period. They normally correct among themselves whoever is practicing wrongly compared to what they have been teaching one another. In addition, untrained TBAs reported receiving training from their mothers-in-law and their parents on delivery practices and taking care of a mother who has given birth. This has been an intergenerational transferred kind of training.

TBAs also reported on the delivery training that they got many years ago that was organized by the health-care system. Their comments also revealed that the training they got did not cover all TBAs. Trained TBAs had an opportunity to grasp some facility activities as they are close to health facilities and they interact with health workers within the health-care facilities. Trained TBAs responded differently saying that they have been trained on delivery matters by the health-care system. Women reported on the lack of training on maternal health issues among their TBAs.

“They (TBAs’ parents) taught me about delivery practices and how to check for pregnancy. In my case, when I was pregnant I checked myself. I put my fingers in and checked the condition of the baby in the womb. That’s how my parents taught me.” (Untrained TBA No. 5)

“I went to a seminar on delivery practices, eeeh! Women have to come to the health facility and not have home deliveries.” (Trained TBA No. 2)

“The TBAs have got no formal training.” (Client of untrained TBA No. 5)

Both trained and untrained TBAs reported on the availability of the government and the health-care systems in restrictions of delivery practices among the TBAs. This made the untrained TBAs more worried about maternal health services issues as compared with the formal ones, who appeared to be quite confident due to their acceptance in the health systems. They used to be invited to the health-care facilities and seem not to conduct delivery practices. Untrained TBAs considered themselves to be mistreated and threatened by the formal health system. Women also commented on the restrictions of delivery practices where there are no home services available to the TBAs.

“Nowadays if the woman has a home delivery they take you to gaol and ask for the reason for her home delivery.” (Untrained TBA No. 4)

“Nowadays there are no home services in general, eeeh! We did it previously, but nowadays there are no such services (delivery practices at home).” (Client of untrained TBA No. 4)

## Discussion

In this study we found that there were some favorable rituals, such as caring closely for post-partum women and cooking food for post-partum women practiced by TBAs. However, unfavorable practices were also revealed that could endanger the life of women. Our study also found that, in general, awareness of the benefits of formal post-partum care was low, especially among women participants and untrained TBAs. The relationship between formal health services and untrained TBAs is weak but better for the trained TBAs. Similar findings were also reported in other studies conducted in other parts of the world [6,7].

### TBAs favorable caring practices during post-partum care

These findings indicate that women appreciate TBAs’ post-partum practices, which might have two implications for the improvement of post-partum care. First, TBAs’ closeness to women might become a window opportunity to detect risk signs and refer women with such signs to health facilities for further investigation and/or treatment.

Other studies show similar findings [7,9-12,24], that TBAs meet community needs in supporting women through pregnancy, childbirth and the post-partum periods. By being close to post-partum women and visiting them periodically, TBAs are in an advantageous position to detect complications such as retained products. Our findings align as well with the continuum of care approach, which underlines the importance of care provided at the household and community level for promoting maternal well-being and health and detecting problems [6].

Second, the rituals performed by TBAs during the post-partum period such as sponging the mothers, caring closely and recognizing problems were appreciated by women. Formal post-partum care might integrate some of those rituals as a way of attracting more women, through increasing the cultural accessibility of services. It is, however, important to acknowledge the scarcity of health workers when thinking of integrating more practices as the workforce is already overstretched. The findings are also in line with those of the study by [42,43] that the post-partum period was commonly associated with rituals and practices that were believed to protect and promote the health of the mother and the baby. The childbirth humanization approach [44-46] aims to increase the cultural accessibility of services throughout the course of childbirth, including aspects that are appreciated by communities and that might be beneficial during delivery – or at least not harmful. It has been applied mainly to delivery services, for example offering delivery in other positions, namely squatting, adapting illumination, clothes or allowing the use of herbal teas. Some studies show evidence that making services more culturally accessible for communities increases their use [47]. Researchers revealed that a high rate of home births in different settings can be due to the poor quality of health services being offered, a lack of resources at the health centres, and a lack of intercultural understanding between the indigenous patients and the health workers [48,49]. A study conducted in Latin America revealed that poor access to health care and preventative services is the norm for the indigenous population and the services that do exist are culturally inappropriate [50]. Enhancing favorable cultural accessibility to post-partum services that might not compromise the health of women might also increase their utilization.

### Perceptions and practices of TBAs on provision of care during complications and its implication to women’s health

In this study when faced with complications, TBAs were not only referring women to health-care services but also trying to handle some complications and their lack of training and lack of use of aseptic techniques increases the risk of infection. At the same time, while they try to handle complications they might prevent women from timely accessing health-care services that can effectively deal with such complications. While some studies point out that TBA training would reduce the first delay [51-53], others highlight that training of TBAs could prevent mothers from using formal health-care services [54] and thus increase maternal mortality and morbidity. In fact, the Government of Tanzania is no longer focusing on the TBAs delivery practices for the reduction of maternal morbidity and mortality, it instead focuses on TBAs counseling and referrals to formal services [55]. Studies in Tanzania indicated that TBAs exist because access to formal health-care services is not ensured for all mothers [56,57]. For example, for women who cannot access the health-care services, their choice was to deliver under TBAs or at home. Similar findings were also reported in Cameroon [58].

Since utilization of post-partum services at the formal health-care services in Dodoma seems to be poorer than utilization of TBAs, it might be useful to consider them in order to improve maternal health care. However, how best to do this in order to improve, and not hinder, women’s use of formal health-care services remains unclear.

### Perceptions of TBAs on women’s formal post-partum care use

A mother who has given birth is supposed to visit on a follow-up basis three times up to 42 days. This helps to monitor her health, detect complications and provide advice related to maternal health issues [1,59]. Whilst some components of post-partum care can be provided in the household and/or community such as education on family planning and breastfeeding, others have to be conducted or at least supervised by skilled health attendants, i.e vaccination, provision of contraceptives. This becomes especially relevant in terms of diagnosis and treatment of complications such as sepsis [3,60]. TBAs can become a link to the health facilities.

This study found that TBAs remain close to women during the post-partum period and their advice might be highly valued by those women, including advice on attending formal post-partum care. Other studies have also found that TBAs’ advice is highly valued by the women they attend [61-63]. However, our study also points out that overall, TBAs perceived that post-partum check-ups were beneficial only for the baby, or when complications arose. Nonetheless, this perception reflects the general practice in the health-care system, where for many years in Tanzania there has been little emphasis on care of the mother after child birth and the main focus has been on the baby with a well-structured plan of visits for health checks and immunization. Moreover, TBAs were uncertain regarding the quality of care provided, which aligns with findings from a previous study exploring women’s perception of maternal health-care services in this area [64].

This implies that TBAs, especially untrained TBAs, may not recommend formal post-partum care and may refrain from referring women to formal health-care services. This can cause delays in reaching health-care services, which could increase morbidity and even mortality in the post-partum period. While one study reported that TBAs in Democratic Republic of Congo have been successfully trained to refer seriously ill mothers [65], other studies revealed that the TBAs integration may have low impact on maternal mortality [66-68]. The national health policy does not support TBAs attending deliveries, but encourages them in engaging in other important roles such as counseling, detection of danger signs and provision of adequate referrals to formal health-care services [4].

TBAs are not the only ones with low awareness regarding the benefits of post-partum care. Post-partum care remains the most neglected stage in the continuum of care worldwide [3,43,69]. In Tanzania, a study conducted by Mbekenga et al. found that post-partum health concerns included a lack of information on basic aspects of care for the mother and infants after childbirth [25].

### Perception of TBAs in their link to health systems and post-partum training

This study shows that the link between untrained TBAs and the health system was weak and cooperation between them was poor. Untrained TBAs perceived that, although they escorted women with complications to health facilities, health providers neither appreciated their efforts nor collaborated with them. They also criticized the programs that penalized them for participating in deliveries. The trained TBAs commended the use of formal health-care services. However, they seemed to be much fewer than the untrained ones.

Training a larger number of TBAs on post-partum referrals can increase women’s access to formal post-partum care services, which can contribute to improve the health of the mothers and their neonates [70]. In a study conducted in Tanzania, it was shown that involving TBAs in the “Safe Motherhood” promotion made them active promoters of skilled birth attendance [53]. Through such programs, TBAs refrained to conduct home deliveries and increased referrals to formal health-care services [53]. Linking the community and health-care system might bring more positive outcomes since research shows that poor connections and weak referral links between community and facility can limit care provided to the most need [6]. Our findings support the need to have linkage among homes, families and community and this is also the aim of the continuum of care approach [6]. However, the link should be clearly described and emphasis should be elaborated on the need to use formal health-care services rather than conducting the services at home which could have poor health out comes to the mother. Even so, all efforts to improve the linkage between the families, communities and the health care system will be insufficient if not coupled with improvement of quality of care in health facilities, infrastructure (roads and means for transport) and access to skilled health care.

Studies elsewhere point out that, when TBAs are trained, their ability to adopt the required improved practices is not universal and the extra confidence they gain after being trained might lead to higher incidence of dangerous procedures and delays in referring women for specialized EmOC services [14-16,71]. However, excluding TBAs from maternal health-care policies and focusing on EmOC has not been able to reduce maternal mortality [72-74]. Well sensitized and trained TBAs can play a key role in reducing maternal mortality and morbidity. For example in the Kigoma region an intervention to train and involve TBAs in detecting post-partum hemorrhage and managing it through the use of misoprostol showed positive results [75,76]. Additionally, a study done in low resource settings including Tanzania and Angola, indicated that training TBAs in the correct use of misoprostol administration reduced PPH, influenced referrals to the formal health-care services and reduced cost of expenditures on health-care services [77]. The initiative to pay TBAs for referrals and to engage TBAs in training activities is also a way of collaboration that has been tried in Philippines [78].

In fact, the most recent continuum of care approach aims for the integration of community, TBAs and formal health providers in a coordinated response in order to improve maternal health [5]. Further, the recent Tanzania post-partum care guidelines also aim for integration of community in family support and improving the referral system as part of the guidelines [59]. The position of TBAs as key stakeholders in the community, especially in hard-to-reach areas, is still a challenge. Studies in Tanzania found that the training of TBAs in maternal health and strengthening the collaboration between TBAs and the health system can be useful [31,79,80]. This also aligns with the national health policy [27]. Health systems working in collaboration with TBAs might increase post-partum utilization and consequently improve maternal health, especially in rural areas and other hard-to-reach areas, where there may be no health facilities in close proximity.

## Limitations of the study

In our study it was difficult to get information due to ongoing restrictions of the delivery practices of TBAs in the community, the untrained TBAs were a bit reserved to provide information as they feared they might be sued due to what they were reporting. A proper methodology to investigate information about TBA practices is needed. We therefore recommend ethnographic research [81], where the researcher requires significant immersion in the study site to allow the investigation to comprehend the ways of life of the TBAs such as their practices with post-partum women. In addition, the recruitment of women through their untrained TBA directives and the recruitment of trained TBAs through the health facility in-charges could have led to social desirability bias. We would also like to acknowledge that recall bias might have been a problem among women whose deliveries took place some years back. All in all, our findings were both positive and negative responses, which reflect the minimal risks of getting biased information. Two issues that were not explored in this study and that deserve further research were that of more in-depth exploration of TBAs practices during PPH and informal payments to TBAs. In addition, we did not include trained TBAs’ clients, due to practical reasons: since these TBAs are not supposed to attend deliveries and finding women who stated that they have been attended to by trained TBAs or using trained TBAs was difficult.

## Conclusions

This study found that the TBAs conduct close follow-ups and some of their practices were appreciated by women. However, the fact that they were trying to manage certain post-partum complications despite their lack of training might put the health of the women at risk. This could be further aggravated by the fact that the linkage between TBAs and formal post-partum services was very weak.

These findings point out the need to increase the quality of the TBA services, especially in terms of prompt referral through provision of training, monitoring and supervision of the TBA practices. Improving the quality of formal post-partum services and promoting the use of post-partum check-ups among women is also needed.

The Ministry of Health and Social Welfare and other stakeholders need to invest in raising awareness on the importance and availability of post-partum services both through the training of TBAs, as well as through mass campaigns so that women are encouraged to use them. Ensuring cultural accessibility of formal post-partum health-care services might also increase women’s acceptance of these services. Finally, efforts should also be made to explore ways to improve collaboration between TBAs and the health-care system through mapping the situation (how many TBAs are there, where are they located, what do they do), sensitizing and training TBAs on facility based post-partum care service use and encouraging referrals.

Furthermore, efforts to improve post-partum care service utilization should be supported by improving access to transport, road infrastructure and the quality of care provided so that women and their families feel welcomed and receive reasonable value for the time invested in attending the services.
